# Referrals of women with a family history of breast cancer from primary care to cancer genetics services in South East Scotland

**DOI:** 10.1038/sj.bjc.6601348

**Published:** 2003-10-28

**Authors:** H Campbell, S Holloway, R Cetnarskyj, E Anderson, R Rush, A Fry, D Gorman, M Steel, M Porteous

**Affiliations:** 1 Department of Public Health Sciences, University of Edinburgh Medical School, Teviot place, Edinburgh EH8 9AG, UK; 2 Department of Clinical Genetics, Molecular Medicine Centre, Western General Hospital, Crewe Road South, Edinburgh EH4 2XU, UK; 3 Edinburgh Breast Unit, Western General Hospital, Crewe Road South, Edinburgh EH4 2XU, UK; 4 SE Scotland Breast Screening Service, Ardmillan House, Ardmillan Terrace, Edinburgh EH11 2SL, UK; 5 Cancer Research UK, Edinburgh Oncology Unit, Western General Hospital, Crewe Road South, Edinburgh EH4 2XR, UK; 6 Lothian NHS Board, Deaconess House, 148 Pleasance, Edinburgh EH8 9RS, UK; 7 School of Biology, University of St Andrews, St Andrews, Fife KY16 9TS, UK

**Keywords:** breast cancer genetic risk counselling, service delivery, primary care, deprivation

## Abstract

As part of a cluster randomised trial to assess an alternative model of cancer genetics services, we gathered data on all referrals from general practitioners (GPs) to cancer genetics services in South East Scotland over a 4-year period. The referral rate per 1000 patients rose by 48% from 0.21 in the 2-year period before the trial to 0.31 during the trial. This increase was much greater in the trial group offered the GP clinic service (64% increase compared to a 38% increase in those referred to the regional service). Thus, the offer of a more local service appeared to have a marked effect on GP management of these women. Referral rates to cancer genetics services from general practices varied widely with higher referral rates from practices with more female partners. There was a negative correlation between referral rates and practice area deprivation scores. However, this was not found during the trial in the group which offered clinics in general practice, the provision of clinic appointments nearer to the homes of more socially deprived women resulting in improved access to women from deprived areas. The interaction with the GP appears to be associated with an inappropriate level of interest in and expectation of the appropriateness of genetic testing. The provision of the clinics within general practice did not result in higher levels of confidence among GPs in managing these women.

UK genetic services are based on a network of regional centres offering specialist services (diagnosis, risk assessment, counselling, surveillance and support) to families at high risk of serious genetic disorders. While links with secondary and tertiary specialists are established in most centres, links with primary care are in the process of development (Donnai and Elles, 2001).

Family history is known be a significant risk factor for breast cancer. Awareness of the genetic component of certain forms of cancer and therefore the potential importance of a family history is increasing among the population. This, in turn, has resulted in an increasing number of self-referrals to general practitioners (GPs), and subsequently to clinical genetics services (Campbell *et al*, 1995). A survey of 22 regional cancer genetics services in the UK in 1998 reported that the predominant users of these services were women with a family history of breast cancer (Wonderling *et al*, 2001).

Of women who are diagnosed with breast cancer, about 10% report of having a family history of the disease. We have recently found, in a large population-based survey of family history of breast cancer, that 52% of adult women have at least one first- or second-degree relative with breast cancer. Thus, all GPs will have many patients with first-degree relatives with breast cancer and many of these patients are likely to seek counselling and advice regarding their level of risk.

The aims and objectives of genetics services responding to the increasing public recognition of family history of cancer have been described in detail (Ponder, 1994). The provision of cancer genetics services has been seen as one of a number of cancer-control strategies located in primary care (Austoker, 1994). In South East Scotland, a multidisciplinary clinic offering specialist cancer genetic risk counselling and screening to women with a family history of breast cancer has been held in the regional breast-screening centre in Edinburgh since 1992. This clinic accepted direct referrals from GPs or other hospital consultants.

We had previously proposed an alternative model of cancer genetics services (Campbell *et al*, 1995), whereby genetics nurse specialists could offer risk estimation based on an assessment of the family history of cancer within clinics held in GP locality areas. This would be accompanied by appropriate counselling for those whose empiric risk was not significantly increased and by immediate referral to regional specialist services for those at higher risk. It was hoped that this would provide improved support to primary care and more appropriate services for those at lower risk while encouraging more cost-effective use of specialist resources for those at increased risk of developing breast cancer.

We carried out a cluster randomised trial of this new model of service delivery comparing it to the existing multidisciplinary specialist service. As part of the data collection for this trial, we gathered structured data on all GP referrals to cancer genetics services in the South East of Scotland over a 4-year period. In this report, we present data on rates of referral of women to regional cancer genetics services for further assessment of their family history of breast or breast/ovarian cancer before and during the period of the trial. We identify and discuss factors that influence these referral rates and report the views of GPs about their role in the management of women with a family history of breast cancer and their attitudes to the services available to them.

## METHODS

### Referral by GPs to cancer genetics services

We defined a study population as the patients registered with general practices within the catchment region of the SE Scotland Cancer Genetics and Breast services. We calculated the total number of referrals from all 203 general practices in this region, which were approached to take part in the study during a 24-month period before the trial started and the 21-month period of the trial. The total number of patients on the lists of all these general practices was 1 221 261 at the beginning of the trial. The referral rate was estimated by


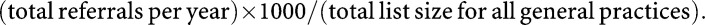


Thus, the referral rates to cancer genetics services before and during the trial were taken as the number of women referred per 1000 patients on the general practice lists during the periods from 1 May 1995 until 30 April 1997 and from 1 March 1998 until 30 November 1999, respectively. Referral rates for individual general practices were calculated in a similar fashion.

The Carstairs deprivation score that is based on postcode of residence was used as a measure of social deprivation of patients registered with general practices (Carstairs and Morris, 1990) with high positive deprivation scores being indicative of greater deprivation. For general practices in Lothian Region, deprivation scores for all patients registered with each practice were averaged to give a mean score for each practice. For general practices in Fife and Borders regions, individual scores were not available so we adopted the score for the postcode of the surgery address to represent the practice.

In order to assess whether differences in referral rates might be in part due to less selective referral criteria used by practices with higher referral rates (resulting in a greater proportion of women with a low risk of developing breast cancer), we classified general practices into four groups according to their referral rate during the trial. In each of these four groups, we calculated the proportion of referrals that were estimated to be at high, moderate and low risk based on an assessment of family history information given in the GP referral letter, thus reflecting the information available to the GP at the time of referral. In a few cases, the information in the GP letter was insufficient for an accurate risk assignment and so these women were omitted.

Women were asked if they had taken the first step by asking to be referred or if this had been suggested to them by their GP, a hospital doctor or another medical professional. Those who said that they had taken the initiative themselves were asked if this was because of their own concern, the suggestion of another family member, because of something in the press or for another reason.

### Summary of the conduct of the trial

Ethical approval for the study was obtained from the local research ethics committee. An invitation to take part in the trial was sent to all general practices in Lothian (*n*=125), South West Fife (*n*=54) and Borders (*n*=24) National Health Service (NHS) Boards in South East Scotland. A total of 170 practices (84%) agreed to take part, 23 (11%) declined and 10 (5%) did not reply. This meant that 725 of the 828 (88%) GPs in practice across these three NHS Board areas agreed to refer patients into the trial. Practices were randomly assigned to either arm of the trial using a minimisation technique (Pocock, 1983) to ensure that the two groups were balanced for size of practice, historical referral rate to cancer genetics services and social deprivation index.

During the period from March 1998 to November 1999, any woman referred from participating GP practices to the regional clinical genetics department for breast cancer genetic risk estimation and counselling was invited to take part in the trial. To be eligible for the trial, women had to live in the region, be able to give informed consent and to complete a baseline questionnaire. Women were asked to record their date of birth, marital status and educational level on the baseline questionnaire. Information about the category of risk to which each woman had been assigned was derived from the clinical records.

Women who were symptomatic or had been diagnosed with breast and/or ovarian cancer or who had previously consulted another clinic about their family history of cancer were excluded from the trial. Those who were ineligible to participate, nonresponders and those who did not consent to participate in the trial were offered an appointment by the regional genetics service. The service offered to women who were enrolled in the trial was dependent on the arm of the trial to which their GP practice had been randomised.

Women referred by the first (‘regional clinic’) group completed a family history form and those considered to be at increased genetic risk (i.e. in the ‘moderate-risk’ or ‘high-risk’ categories) received the existing service that comprised an appointment to see a consultant geneticist and breast surgeon at a regional centre. Women referred by the second (‘community clinic’) group were seen at one of several clinics held in a community setting relatively near the woman's own general practice.

At the community clinic, the genetics nurse specialist ascertained the woman's family history of cancer and compiled a family tree. This information was compared to published criteria to determine whether she was at increased risk. When an adequate risk assessment could not be made during the appointment, further information and/or confirmation of relatives' diagnoses were obtained from the woman or medical records or from cancer registry data, before the woman was informed of their risk by letter. Women whose risk of breast cancer was estimated not to be increased over that of women of a similar age in the general population (i.e. in the ‘low-risk’ category) were offered information and reassurance and were discharged from the clinic. These women and their GPs were sent a letter reaffirming their low-risk status and summarising the issues discussed at the appointment. Women whose risk was estimated to be increased over that of women of a similar age in the general population were offered an appointment at the regional centre with a consultant breast surgeon and genetics nurse specialist. Further details of the trial interventions are given in a related publication (Fry *et al*, 2003).

Psychological distress and cancer worry were measured by the general health questionnaire (GHQ 30) (Goldberg and Williams, 1988) and the Cancer Worry Scale (Watson *et al*, 1998) as described by Fry *et al* (2003).

### Questionnaire survey of general practices who participated in the trial

All GPs who referred women during the study period received a questionnaire asking their views about various aspects of the management of women with a family history of breast cancer.

### Statistical methods

*χ*^2^ tests were used to compare the distribution of sources of referral before and during the trial, the risk statuses of women who were referred by practices with different referral rates, and the responses of women who had or had not been given information by their GPs. Comparisons were made between referral rates within trial groups using the paired *t*-test. Comparisons of the number of practice members and female practice members were made using the Spearman rank correlation coefficient since these were not normally distributed. Pearson's correlation coefficients were calculated between locality (Carstairs) deprivation scores and referral rates before and during the trial for all Lothian practices.

## RESULTS

### Referral rates to cancer genetics services

#### General practices approached to take part in the study

A total of 203 practices in the Lothian, Borders and South-West Fife regions of Scotland were approached to take part in the study. Some 170 (84%) agreed to take part, 23 (11%) refused and 10 (5%) did not reply.

#### Changes in referral rates to cancer genetics services over time

The referral rate (and 95% confidence interval) per 1000 patients on the GP lists rose from 0.21 (0.19–0.24) in the 2-year period before the trial to 0.31 (0.28–0.34) during the trial. Thus, there was a 48% increase in referral rate over this period of approximately 2.5 years, a highly significant difference (*P*<0.001).

#### Change in referral behaviour of GPs

Prior to the study, many asymptomatic women with a positive family history were referred to the Edinburgh Breast Unit (symptomatic breast clinic) and then referred on, after receiving a mammogram and an appointment with a breast surgeon, to the cancer genetics clinic. In order to assess if there had been a change in this pattern of referral during the period of the study, we compared the referral sources of this group of asymptomatic women (with a positive family history of breast cancer) before and during the trial. The letter sent out at the start of the trial to all GPs requesting that referrals be directed to cancer genetics services rather than to symptomatic breast services was successful, with the proportion of referrals from the symptomatic breast service falling from 24.6% before the trial to 14.5% during the trial. There was a highly significant difference between the sources of referral before and during the trial (*P*< 0.001).

#### Relationship between referral rate and risks of women referred

Of the general practices that agreed to take part in the study, 30 referred no women during the study period. Annual referral rates during the trial for other practices varied by more than 30-fold, ranging from 0.05 to 1.66 per 1000 registered patients. The proportions of referrals that were estimated to be at high, moderate or low risk (based on information in the GP referral letter since this was the information available to the GP at that time) by four strata of general practice referral rates are given in Table 1

**Table 1:** Numbers (percentages) of referrals classified as high, moderate and low risk of breast cancer (due to family history based on national criteria) by practice groups with differing referral rates (annual rate per 1000 women on general practitioner list)

		**Risk classification following national guidelines**			
**Referral rate during trial**	**Number of general practices**	**High risk**	**Moderate risk**	**Low risk**	**Total referrals**
⩽0.19	41	7 (11.1%)	48 (76.2%)	8 (12.7%)	63
0.20–0.33	28	11 (13.3%)	55 (66.3%)	17 (20.5%)	83
0.34–0.54	39	26 (12.1%)	133 (62.1%)	55 (25.7%)	214
⩾0.55	31	23 (12.8%)	116 (64.8%)	40 (22.3%)	179
					
Total	139	67	352	120	539

. This shows that although general practices with the lowest referral rates referred a smaller percentage of low-risk women, there was no statistically significant trend in the proportions of risk classifications across the referral rate strata. Thus, we found no strong evidence that higher referral rates reflect the use of less selective referral criteria by these general practices.

#### Relationship between referral rates and number and sexes of partners in the practice

Of the practices that had agreed to take part in the study, 15% had no female partners, 67% had one to two female partners and 19% had three or more. Neither referral rate before nor during the trial was correlated with the number of partners or the number of male partners in the practice. However, there is a small but significant correlation between both referral rates and the number (Spearman rank correlation coefficient 0.24, *P*<0.002; 0.23, *P*<0.003) and proportion (Spearman rank correlation coefficient 0.22, *P*<0.005; 0.17, *P*<0.02) of female partners in the practice before and during the trial, respectively.

#### Comparison between referral rates in the two trial groups

Table 2

**Table 2:** General practitioner (GP) referral rates (per 1000 patients on GP list per year) to cancer genetics services before and during the trial (mean and 95% confidence limits)

**GP group**	**Before trial mean (95% CI)**	**During trial mean (95% CI)**	**Percentage increase in referral rate**
Regional group	0.21 (0.17–0.26)	0.29 (0.23–0.34)	38.1
Community group	0.22 (0.17–0.26)	0.36 (0.29–0.43)	63.6

gives the mean referral rates before and during the trial-by-trial group. There was a statistically significant increase in referral rate between the two time periods in both trial groups (regional group *P*<0.01; community group *P*<0.001). This increase was greater in the community clinic trial group (64% increase compared to a 38% increase in the regional group over rates before the trial).

#### Relationship between referral rates and locality deprivation scores of general practices

We calculated the correlation between the locality (Carstairs) deprivation score and referral rates before and during the trial for all Lothian practices (whether or not included in the trial) to investigate whether general practices serving women who were more socially deprived would have lower referral rates than those serving less socially deprived women. The correlation coefficients were −0.26 (*P*<0.01) with referral rates before the trial and −0.13 (not statistically significant) with referral rates during the trial, suggesting that the tendency for practices serving less deprived areas to have higher referral rates was reduced during the time of the trial. Table 3

**Table 3:** Correlations between general practitioner-referral rates and locality (Carstairs) deprivation scores (higher score in more deprived areas) by trial group

**Trial group**	**Correlation with referral rate before trial**	**Correlation with referral rate during trial**
Regional group	−0.26	−0.28
Community group	−0.23	−0.01

shows that the relationship between referral rate and locality deprivation score in general practices is influenced by the provision of community clinics. There was a negative correlation between referral rate and locality deprivation score both before and during the trial in the regional trial group. This indicates that practices in areas of lower social deprivation had higher referral rates. The same was true of the community trial group before the trial but during the trial the correlation was almost zero. Although the difference in referral rates between the two groups did not reach statistical significance, the lack of change in the regional but not the community trial group is striking (Table 3).

#### Patient's role in the referral by the GP

In all, 43% of women indicated that they had asked to be referred (in contrast to the others for whom referral was suggested to them by their GP or another medical professional). Two-thirds of these women noted that this was based on their own concern with the others requesting referral at the suggestion of another family member. Younger women were more likely to have taken the initiative to request referral (50% of women under 40 years compared to 31% of those 40 years or over, *P*=0.001, *χ*^2^ test).

Of those that stated that their GP (or other medical professional) had initiated the referral, only a third stated that they had specifically enquired about their family history of cancer; for over one-half the suggestion of referral had taken place when they had seen the doctor about another matter. There was no significant difference in educational status, perceived level of risk or cancer worry between the two referral groups.

### Women's views of information given to them by their GP prior to referral

Women enrolled in the trial were asked questions about whether any information had been given to them by their GP about their family history of cancer prior to the referral. About 40% of women had received no such information. In total, 50% of those who had received information reported that they found it to be very helpful or helpful. Women were then classified into two groups according to whether or not they had received information.

The only statistically significant difference between the two trial groups (after correcting for the number of comparisons made (by the Bonferroni method) related to views on genetic testing. In all, 90% of those who had received information from their GP thought that it was very important or quite important to have genetic testing compared with 73% of those who had not received information from their GP (*P*<0.01). Similarly, 71% of the group who had received information from their GP stated any information from a genetic test would be very important compared with 56% of those who had not received information from their GP, but this difference was not statistically significant.

### General practitioners' views about the management of women presenting with a family history of breast cancer

All GPs who referred women during the period of the trial received a questionnaire asking their views about various aspects of the management of women with a family history of breast cancer. Completed questionnaires were received from a total of 129 GPs in the regional group and 115 GPs in the community group.

#### General practitioners' confidence in fulfilling their role in cancer genetics services

Table 4

**Table 4:** Percentages of general practitioners expressing their degree of confidence in fulfilling various roles in the management of women presenting with a family history of breast cancer

	**Not confident or a little confident**		**Moderately confident**		**Confident or very confident**	
	**Regional**	**Community**	**Regional**	**Community**	**Regional**	**Community**
Taking detailed family history	25.6	21.8	38.8	35.7	34.9	42.6
Calculating the risk	89.9	87.8	10.1	10.4	0	0.9
Counselling on risk	83.0	75.7	13.2	20.9	3.9	2.6
Providing emotional follow-up support	17.1	23.5	42.6	39.1	40.3	37.4
Providing regular clinical examination	29.4	21.8	37.2	40.9	33.4	37.4
Teaching breast self-examination	14.0	13.9	33.3	33.0	52.8	53.0
Discussing need for mammography/colonoscopy	28.7	33.0	42.6	41.7	28.7	25.2
Deciding whether patient should be referred to regional genetic clinic	41.1	45.3	50.4	38.3	8.5	15.7

shows the percentages of GPs having various degrees of confidence in handling various aspects of the management of these women. General practitioners in both groups were most confident about taking a family history, providing emotional follow-up support and regular clinical examination and teaching breast self-examination. They were much less confident about calculating the risk and counselling on the basis of this and less confident about discussing the need for mammographic screening and deciding about whether a patient should be referred to the genetic clinic. There were no marked differences between the two trial groups suggesting that the limited number of contacts between the GPs and the genetics nurses who staffed the community clinics was insufficient to alter GP confidence in the management of these women. There was little evidence of GPs taking advantage of the presence of the nurse within the practice to discuss genetics issues.

#### General practitioners' attitudes to genetic counselling and active screening for family history of breast cancer

Over 90% of GPs agreed or strongly agreed that cancer genetic counselling has a useful role for women with a family history of breast cancer, and over 85% agreed or strongly agreed that mammography has a useful role for those at increased risk. In contrast, however, only 30% agreed or strongly agreed that GPs should actively identify those from their lists who might be eligible for genetic counselling.

#### General practitioners' views on their information and training needs

General practitioners' views on the potential utility of various forms of support from regional clinical genetics services in helping them deal with their increased workload are given in Table 5

**Table 5:** Percentages of general practitioners' stating views on the usefulness of various forms of support for the management of women with a family history of breast cancer

	**Not useful or a little useful**		**Quite useful**		**Very useful**	
	**Regional**	**Community**	**Regional**	**Community**	**Regional**	**Community**
Interactive computer program	34.1	37.4	47.3	36.5	17.1	22.6
Referral guidelines	9.3	6.1	45.0	43.5	45.7	48.7
Local clinics offering genetic counselling	27.2	20.9	44.2	42.6	28.7	33.9
Direct access to medical genetic screening	36.4	38.2	41.9	38.3	20.2	19.1
Training for yourself in genetic counselling	57.4	56.5	29.5	30.4	13.2	10.4
Training for other primary care staff in genetic counselling	64.3	56.6	27.9	30.4	7.0	11.3

. Referral guidelines were considered to be the most useful support, with over 90% of GPs regarding them as useful or very useful. Local clinics offering genetic counselling were regarded by one-third as very useful. There was less enthusiasm for interactive computer programs or direct access to screening. Over 50% of GPs stated that training in genetic counselling for themselves or other practice staff would be not at all useful or only a little useful. It is possible, however, that this was due to their interpretation of the term ‘genetic counselling’ and that a higher proportion of GPs would favour some form of training to improve their skills. There were no differences in responses between GPs in the two trial groups.

### General practitioners' views on trial interventions

About two-thirds of GPs (62% of regional group GPs and 65% of community group GPs) noted that they were confident or very confident about their women being seen by a genetic nurse specialist or genetic associate rather than by a consultant geneticist. A similar proportion (61% of regional group GPs and 64% of community group GPs) noted that they were positive or very positive about having genetic clinics in the community. When asked whether they would prefer community clinics run by nurse specialists or regional hospital-based genetic clinics about one-third (36% of regional group GPs and 34% of community group GPs) favoured the former and one-half (51% of regional group GPs and 50% of community group GPs) the latter, the remainder being undecided.

General practitioners found the structured summary letter the most useful aspect of the service followed by the provision of local community-based clinics (rated as useful or very useful by 44.4 and 37.4%, respectively). Other items (practice talks given by genetics nurse specialists and telephone advice) were rated as useful or very useful by less than a quarter of the GPs. General comments made by GPs included that they had made little use of the service and that women were less likely to default from appointments at community clinics.

## DISCUSSION

### Specialist outreach clinics

Specialist outreach clinics in general practice increased throughout the mid-1990s, reflecting a desire within the National Health Service to move towards closer integration of primary and secondary care services (Bailey *et al*, 1994). Within clinical genetics, it is recognised that this has the potential to lead to improved equity of access to high-quality regional services (Donnai and Elles, 2001), although this has not been formally evaluated. Within primary care, there has been a call to develop strong links with regional genetics centres. This has stressed the need to provide accurate information and support for the primary care team and to undertake some genetic risk assessment and counselling and facilitate appropriate referral within primary care (Kinmonth *et al*, 1998).

There have only been a limited number of thorough evaluations of outreach services across various disciplines. These have reported improved waiting times, patient satisfaction and convenience to patients but have noted less efficient use of specialist time, limited interaction between primary care and specialist staff and concerns about access to these services not being uniform throughout a region (Bowling *et al*, 1997).

### Prevalence of family history of breast cancer and referral rates to cancer genetics services

We recently carried out a large cross-sectional survey of 13 155 patients registered with GPs in Scotland (Wallace E *et al*, personal communication). This found that a GP with an average caseload of 1700 patients would have 140 patients with a family history of breast, colorectal or breast/ovarian cancer, and of these 10 would meet national criteria for referral for risk assessment. Reported referrals to regional services for consultation regarding a family history of breast cancer suggested a referral rate of about 0.25 referrals per 1000 patients per year, consistent with the referral rates found in this study. This is based on referral of patients who presented spontaneously to their GP and not on any form of active surveillance by GPs of family history of cancer within their practice population.

During the study, there was nearly a 50% increase in referral rate, compared with 1–3 years prior to the study and a greater proportion of referrals came directly from GPs. At the start of the study period, local protocols, based on UK recommended guidelines for the primary care management of people with a family history of breast cancer, were developed together with GP representatives and Health Board guidelines groups. These local protocols were disseminated to all GPs in South East Scotland. In addition, all GPs received a biannual genetics update newsletter during the course of the study. These factors are likely to have contributed to the increase in referral rate.

A striking finding was the substantially greater increase in referral rates from pretrial levels in the community clinic trial group compared to the regional group (64% increase compared to a 38% increase). Thus, in addition to the underlying general increase in referral rates, the establishment of community clinics resulted in a change in referral behaviour that resulted in a further increase in referral.

Despite the increase in referral rates that we recorded over this 3-year period, these rates are considerably lower than those (15 out of 1000 adult women/year) at which women reportedly raise concerns about family history of breast cancer with their GP (Kinmonth, 2001) suggesting that the GP ‘gatekeeper’ role is an important one.

There was a very wide (more than 30-fold) variation in the annual referral rates. The reasons for this are not clear, but there was a small but significant correlation between referral rates during the trial and the number and proportion of female partners in the practice. Referral rates were greater from those practices with a greater number of female partners and this could be because women with a family history are more likely to seek the advice of a GP, if the GP is female. Thus, if the patient belongs to a practice where it is impossible or more difficult to see a woman, they may be less likely to seek advice. Alternatively, it could be that female GPs are more likely to refer women for genetic counselling about breast cancer risk than male GPs.

We postulated that higher referral rates may reflect the use of less rigorous selection criteria by some GPs. For example, this might have occurred because of their knowledge that selection, according to risk, for referral to the breast-screening service would be performed subsequently by the genetic nurse who interviewed the woman at the locality clinic. However, we found no evidence that this had occurred.

We interpret our finding as follows. Family history of breast cancer is common and many women consulting their GP may mention this at the time of consultation (Kinmonth, 2001). We found that younger women are more likely than older women to raise this concern and request referral to specialist services. General practitioners are operating at different thresholds at which they take action on these concerns. We have already shown that about 10% of women with a family history of breast cancer had been referred to a specialist service by their GP (Wallace E *et al*, personal communication). Once the decision to respond to this concern is made by the GP, similar referral criteria are applied. Currently, a more active surveillance of family history of cancer in primary care (which would result in a much higher referral rate) is not recommended in national cancer genetics guidelines nor is it supported by GPs (in this survey only 30% agreed that GPs should actively identify those from their lists who might be eligible for genetic counselling).

### Relationship between deprivation and referral rates to cancer genetics services

There is an extensive literature that confirms that people from different socioeconomic groupings consume health care in different ways. This almost invariably shows that more deprived people have worse health and have greater need for health care (Balarajan *et al*, 1992; Eachus *et al*, 1996). It has been found that 15–20% of the overall variation seen in the overall GP referral rates (to all services) can be explained by deprivation (Hippisley-Cox *et al*, 1997). Disadvantaged groups have also been shown to be less likely to attend for breast and cervical screening preventive services (Eachus *et al*, 1996).

Provision of health care should be primarily determined by need. However, access to secondary care has been reported to be selectively poorer in deprived groups (Chaturvedi and Ben-Shlomo, 1995). In this study, the correlation coefficient between deprivation score and referral rate remained unchanged in the regional group: −0.26 before the trial and −0.28 during the trial. Thus, prior to and during the study in the regional group, there was a tendency for practices serving less deprived areas to have higher referral rates. The differential use of cancer genetics services across deprivation groups may be influenced by factors such as perceived risk and financial considerations (Eachus *et al*, 1996) or by health behavioural factors involving knowledge of importance of family history, how personal risk is perceived and what action is taken to seek counselling for a personal assessment of high risk and the ability to articulate need to health service staff.

However, this relationship changed in the community group with the establishment of new community clinics with the correlation between deprivation score and referral rate before the trial (−0.23) falling to −0.01 during the trial. Thus, the tendency for practices serving less deprived areas to have higher referral rates was no longer found where community clinics were held This is consistent with an interpretation that the provision of clinic appointments nearer to the homes of more socially deprived women and staffed by nurses results in GPs more likely to refer and/or women being more willing to attend these clinics than more distant regional clinics staffed by consultants. In addition, GPs commented that women were less likely to default from appointments at community clinics.

#### Genetic testing

We found that the interaction with the GP was associated with an inappropriate level of interest in and expectation of the appropriateness of genetic testing, with 90% of those who had received information from their GP considering it important or very important compared to 73% of those who had not received information from their GP. While it is possible that this was due to recall bias, we consider this unlikely and suggest that this is worthy of further investigation. Since genetic testing is only appropriate to a very small percentage of women with very high familial risk, it is important that GPs do not foster this level of expectation.

This mismatch in perception about the role of molecular testing between those running cancer genetics clinics and women attending them may need to be addressed specifically in the way clinics are organised in the future and in postgraduate training of GPs.

### Views of GPs on new services and on their role in cancer genetics

In general, GPs were confident about taking a family history, clinical examination and offering emotional support. They were less confident about risk assessment and deciding if mammographic screening was necessary, and most did not see it as their role to identify those from their lists who might be eligible for genetic counselling. Several commented that they and their staff did not have time to take on additional work of this nature.

There were no marked differences between the two trial groups suggesting that the limited number of contacts between the GPs and the genetics nurses who staffed the community clinics were insufficient to alter GP confidence in the management of these women. This was reinforced by the written comments of a number of GPs on the survey questionnaire that they had made little use of the service (the average referral rate was 0.5 referrals per GP per year). It has been noted previously that the limited interaction between primary care and specialist staff jeopardises achievement of one of the central aims of these initiatives – to facilitate integration and overcome barriers between primary and secondary/tertiary care (Bailey *et al*, 1994).

In general, GPs were positive about their patients being seen by genetic nurse specialists and about genetic clinics in the community. However, one-half still favoured hospital-based rather than locality clinics. General practitioners found the structured summary letter the most useful aspect followed by the provision of local community-based clinics. Most GPs regarded the referral guidelines provided by the study as useful. Other items (practice talks given by genetics nurse specialists, telephone advice and trial newsletters) were rated as useful or very useful by less than a third of the GPs.
